# Magnetic Bacterial Cellulose Biopolymers: Production and Potential Applications in the Electronics Sector

**DOI:** 10.3390/polym15040853

**Published:** 2023-02-09

**Authors:** Thaís Cavalcante de Souza, Julia Didier Pedrosa de Amorim, Claudio José Galdino da Silva Junior, Alexandre D’Lamare Maia de Medeiros, Andréa Fernanda de Santana Costa, Gloria Maria Vinhas, Leonie Asfora Sarubbo

**Affiliations:** 1 Centro de Ciências Exatas e Naturais, Departamento de Ciência dos Materiais, Universidade Federal de Pernambuco, Rua Prof. Moraes Rêgo, n. 1235, Cidade Universitária, Recife 50670-901, Pernambuco, Brazil; 2 Instituto Avançado de Tecnologia e Inovação (IATI), Rua Potyra, n. 31, Prado, Recife 50751-310, Pernambuco, Brazil; 3 Rede Nordeste de Biotecnologia (RENORBIO), Universidade Federal Rural de Pernambuco (UFRPE), Rua Dom Manuel de Medeiros, Dois Irmãos, Recife 52171-900, Pernambuco, Brazil; 4 Centro de Comunicação e Design, Centro Acadêmico da Região Agreste, Universidade Federal de Pernambuco, BR 104, Km 59, s/n, Nova Caruaru 50670-901, Pernambuco, Brazil; 5 Departamento de Engenharia Química, Universidade Federal de Pernambuco (UFPE), Av. dos Economistas—Cidade Universitária, Recife 50740-590, Pernambuco, Brazil; 6 Escola Icam Tech, Universidade Católica de Pernambuco (UNICAP), Rua do Príncipe, n. 526, Boa Vista, Recife 50050-900, Pernambuco, Brazil

**Keywords:** magnetic bacterial cellulose, electronics, devices, biotechnology

## Abstract

Bacterial cellulose (BC) is a biopolymer that has been widely investigated due to its useful characteristics, such as nanometric structure, simple production and biocompatibility, enabling the creation of novel materials made from additive BC in situ and/or ex situ. The literature also describes the magnetization of BC biopolymers by the addition of particles such as magnetite and ferrites. The processing of BC with these materials can be performed in different ways to adapt to the availability of materials and the objectives of a given application. There is considerable interest in the electronics field for novel materials and devices as well as non-polluting, sustainable solutions. This sector influences the development of others, including the production and optimization of new equipment, medical devices, sensors, transformers and motors. Thus, magnetic BC has considerable potential in applied research, such as the production of materials for biotechnological electronic devices. Magnetic BC also enables a reduction in the use of polluting materials commonly found in electronic devices. This review article highlights the production of this biomaterial and its applications in the field of electronics.

## 1. Introduction

Cellulose is composed of a linear organic chain; it is the most abundant polymer on the planet and can be obtained from different sources, such as plants, algae and via microbiological fermentation [1]. Bacterial cellulose (BC) is produced from bacteria and microbial consortia in the form of biofilms or pellets, depending on the production conditions under which the microorganisms are subjected. This biopolymer has been attracting interest from researchers due to its versatility, unique characteristics and ease in the production of biofilms. The main differences between BC and vegetable cellulose (VC) are related to the degree of purity and crystallinity. Additionally, VC has impurities such as lignin, pectin and hemicellulose [2,3,4]. Another major difference between VC and BC resides in the production and purification processes, as obtaining BC does not depend on factors such as region, seasonality and long periods of time. Other characteristics that attract more research with this biomaterial are its ability to absorb compounds when wet, the formation of a hydrogel, the nanometric size of its fibres, and biocompatibility and strength, making BC a material with considerable biotechnological potential and several possible applications [5,6].

With all these advantages, BC has been the object of study in several lines of research. However, the production cost is high, mainly due to the substrates used in its composition, such as glucose, yeast extract and peptone, which are synthesized/isolated materials sold at high cost. As a result, researchers have sought to optimize and reduce the cost of BC production with the supplementation of agro-industrial residues in the culture medium [7,8,9,10] and the inclusion of additives and modifications of BC, providing new properties to the biopolymer [11,12,13]. BC has considerable versatility and can be combined with other materials. Applications for these novel materials include the medical, pharmaceutical, food and electronics industries [14,15].

Magnetic materials are widely used, but are produced with chemical compounds and petroleum by-products, contributing to large-scale pollution [16]. To circumvent this problem, researchers have sought to obtain biotechnological materials with magnetic properties, which are also known as smart materials [17,18]. These novel materials have various applications and can be manufactured in different ways. The literature also describes the development of magnetic materials containing BC [19].

The magnetization of BC is achieved by the addition of different types of magnetic particles, such as ferrites, magnetite, and nickel. Magnetic BC is a material with considerable technological potential due to its wide variety of applications, production methods and dopants that can be incorporated, enabling adaptation to any production environment, and meeting the demand for magnetic biomaterials. In view of this potential of magnetic BC, the present review addresses the production of this material and highlights some applications found in the literature, particularly biotechnological applications in the field of electronics, which directly influences all other fields and undergoes constant advancement [20,21].

## 2. Bacterial Cellulose

### 2.1. Characteristics

BC has been explored due to its advantages over VC, such as production in less time and requiring little space. Unlike VC, which has a micrometric structure, BC has a structure of nanometric fibres, a higher degree of purity, as well as greater crystallinity, thermal stability and improved mechanical strength. Other important aspects include biocompatibility (not toxic or harmful to health), high absorption capacity of other substances, and the fact that BC is a renewable, eco-friendly resource, which is of considerable interest to the medical, food and cosmetic industries [3,14,22].

While the production of VC requires a longer time due to the growth period of plants, such as cotton and eucalyptus, which can take months and even years, BC can be obtained in 3 to 14 days. The production of VC also depends on large areas for planting trees and shrubs. The maintenance of the crop is expensive and involves a large volume of fertilisers, water, and pesticides to ensure the growth of the plants. VC requires more complex industrial processes for extraction and purification. In contrast, BC can be produced in containers of different sizes and shapes, without the need for daily growth maintenance methods. Moreover, research points to the possibility of making BC production media cheaper with more economical agro-industrial residues, which do not compete with the food industry, thereby increasing the sustainability of culture production [6,23].

Table 1 presents a comparison of some characteristics of BC and VC.

As VC is a polymer of natural origin, it has several components in its structure (lignin, hemicellulose, pectin, etc.). Because it can be derived from different plant species with distinct fibre properties which depend on seasonal and regional factors, VC has a Young’s modulus of great variation, as shown in Table 1.

Another characteristic of this biopolymer that attracts the attention of researchers is its ability to absorb, retain and release other substances. BC has a hydrogel aspect when hydrated and its nanofibers retain about 90% of its total mass in the form of water [5,30,31]. These properties also make it a perfect scaffold for other materials and substances, opening up a vast field of applicability and opportunities for the production of novel materials and biotechnological products [32].

### 2.2. Production

**Figure 1:**
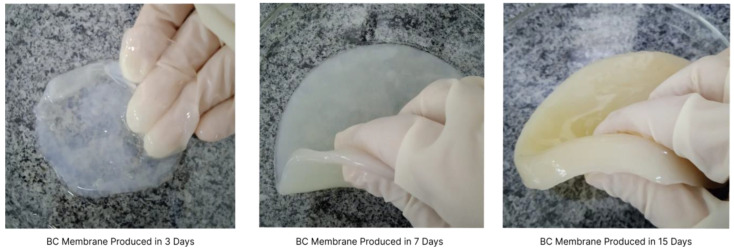
BC films produced after 3, 7 and 15 days.

To produce BC, a culture medium must provide the necessary nutrients for the microorganisms. During the process, microorganisms secrete fibers from metabolized carbon sources. This biopolymer production is carried out on different levels. The bacteria build fibres layer by layer, obtaining a membrane that becomes thicker over its production time [33]. Figure 1 shows BC films produced during 3, 7 and 15 days of fermentation. Some studies indicate that the formation of the biopolymer is a defence mechanism, with the bacteria producing it to protect from UV rays and external contaminants. Other studies suggest that the biofilm is produced to help the bacteria to stay close to the surface and thus acquire more oxygen [34].

Another important point is whether BC production occurs statically or dynamically. The main differences between these modes are the final shape of the biopolymer and the mechanical properties. When production is performed statically, the membrane has a flat appearance and takes on the shape of the vessel in which fermentation took place. With dynamic production, the final product looks like pearls/pellets or dispersed fibres [35,36,37].

**Figure 2:**
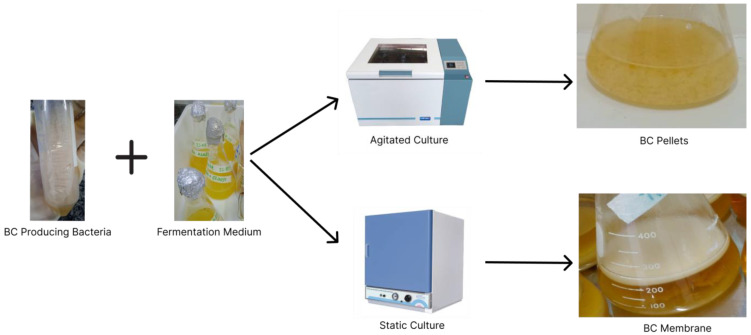
BC production process.

The BC production process can be seen in Figure 2.

Not all bacteria can produce BC. In general, the genera *Sarcina*, *Komagateibacter*, *Pseudomonas*, *Rhizobium* and *Agrobacterium* are good producers. There are also specific culture media for production that must contain sources of carbon, nitrogen, and some mineral salts. The most widely used medium is the one formulated by Hestrin and Schramm [38], which is expensive, as it contains high purity reagents such as glucose, peptone, yeast extract, citric acid, and sodium diphosphate. Some of the residues used come from cereals and grains such as oats, soybeans, corn, wheat, beans, peanuts, coffee, and barley, from fruits and vegetables such as tomatoes, oranges, apples, grapes, bananas, mangoes, coconuts and cocoa, and also residues from other vegetables such as cotton, palm and tea herbs [15,38,39,40]. The use of agro-industrial residues to produce BC membranes is a good option for reducing costs, especially if scaled to industrial manufacturing. It also enables the reuse of materials that would otherwise be discarded [41,42,43].

The improvement of BC films can be achieved not only by production in specific media, but also with the addition of other substances (dopants), giving BC new properties or changing some of its characteristics and transforming it into a matrix for various types of composites and other materials [44,45,46].

After fermentation, BC is purified to remove by-products from the metabolism of microorganisms. This can be achieved by the immersion in a NaOH solution or hot sodium hypochlorite, or by gamma radiation, among other methods [47]. Drying is another action that can be performed after production, which may or may not be necessary depending on the final application of the biofilm. BC can be dried in an oven, outdoors, in freezer dryers, or can be submitted to critical drying processes [48].

### 2.3. Modifications and Dopants

As mentioned above, the properties of BC can be changed and/or modified. There are several ways to enhance and transform biofilms, many of which occur through very simple mechanisms, i.e., several studies take advantage of its great absorption capacity to insert different compounds in this simple way. There is also a wide variety of additives and dopants that can impart different properties to BC. Table 2 shows some dopants reported in the literature and the respective characteristics imparted to BC. There are two means of defining the forms of biomembrane modification: in situ (during fermentation or with the synthesis of particles in its interior) and ex situ (addition of particles after fermentation) [49].

In situ modifications can be achieved with the use of residues as well as fruit and vegetable extracts, or with the addition of others compounds to the culture medium [15,65,66]. There are also in situ processes by which dopants are synthesized within the interstices of the BC fibres. For instance, Usawattanakul et al. [67] synthesized magnetite through an oxidative process within a BC film.

A vast number of methods are used for ex situ modifications (after BC fermentation), such as carrying out synthesis within the BC fibres, immersion in specific solutions for the absorption or adsorption of compounds and incorporation by grinding. The array of doping components that can be added is also enormous, ranging from other polymers, natural or artificial such as VC, polyhydroxybutyrate (PHB) and polyvinyl acetate (PVA), to materials of a natural origin such as plant and fruit extracts, and metals and minerals such as copper, silver, magnetite and ferrite. Some modifications can also chemically change the cellulose structure. In this case, the polarity of BC is modified to insert hydrophobic components in its structure, such as essential oils [65,66].

Another factor that must be considered is its dimensions. BC has nanometric pores [68,69], so nanoparticles must have a better interaction with the biopolymer, lodged in the BC pores. Aiming for a better distribution of the additive’s nanoparticles in the BC, many authors resort to complementary methodologies to avoid particle agglomerations or less concentrated regions in BC, by using equipment such as ultrasound devices, sonicators and ultrasonicators [70,71,72,73]. Another strategy is the use of specific substances. Zhang et al. [64] for example used poly-dopamine to achieve uniformly dispersed metal nanoparticles in a BC-graphene oxide composite. The choice of specific processing methods and selected dopants is important for the creation of a final product, which can be a novel material with different characteristics and properties [46,49]. Among these novel materials made from modified BC, magnetic BC polymers have demonstrated considerable potential for applicability and a variety of production methods. Dopants with magnetic characteristics have been widely researched for the production of BC materials and devices.

## 3. Magnetic Bacterial Cellulose Biopolymers

Magnetism is an inherent characteristic of a material and is related to the movements of its electrons, which can align, generating forces of attraction and repulsion (magnetic moment) in response to external stimuli [74]. When the electrons of a material are aligned in the same direction of an external magnetic field of a certain intensity, the material has reached saturation magnetization. A material’s capacity to maintain electron alignment even after the removal of the external field is called coercivity. Saturation magnetization and coercivity (or coercive field) are important magnetic characteristics to understand the behaviour of a material in relation to how much it can respond to an external field and its performance as a permanent (magnetically hard materials) or temporary (magnetically soft materials) magnet [75,76,77].

Magnetic materials are found in various types of devices with applications in everyday life such as sensors, transformers, magnets, stereos, electronic circuits, data storage systems, etc. [74]. Depending on the desired application, these materials must also have different properties such as biocompatibility for use in medical and pharmaceutical applications, and flexibility, as in the case of malleable sensors [78,79,80]. Several researchers have produced biotechnological magnetic materials, also classified as smart materials [17].

Due to the expansion of studies on biotechnological magnetic materials, many lines of research have turned to the production of magnetic BC. Aspects such as saturation magnetization and coercive field vary according to the production media as well as the types and percentage of magnetic dopants added. An example of this variance is seen in studies that used different production conditions. Salidkul et al. [81] developed BC composites doped with varying percentages of barium ferrite nanoparticles at a ratio of 20:1 g (BC:BaFe_12_O_19_). The authors achieved saturation magnetization of approximately 20.3 emu/g and a coercive field of approximately 5.34 kOe. In the study by Chanthiwong et al. [82], BC films were doped with maghemite and magnetite through in situ co-precipitation, immersing the BC in the reagents. Saturation magnetization of the materials ranged from approximately 25.3 to 57.0 emu/g, with a coercive field close to 0 kOe. The authors designated their materials for different applications that match the magnetic characteristics inherent to the materials produced. Chantiwong et al. [82] suggested magnetic BC for adsorbents in the medical field, while Salidkul et al. [81] suggested their material for electromagnetic adsorbents and data recording systems due to its high coercive field. The comparison of these studies demonstrates that each selected method and dopant has an impact on the final magnetic properties of the material produced and, according to this variation in properties, the materials can be used for several application possibilities.

**Figure 3:**
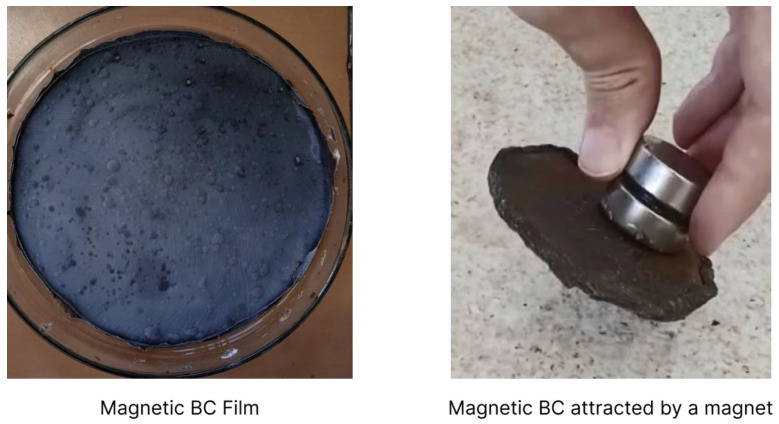
Magnetic BC: film produced through in situ co-precipitation of magnetite in BC fibres.

The visual characteristics of BC also vary according to the types and percentages of dopants. Figure 3 shows a BC doped with magnetite via in situ co-precipitation that was immersed in solutions with the reagents, and acquired a dark colour in shades ranging from graphite grey to black.

Zhang et al. [29] obtained a lighter, more translucent colour due to the acid hydrolysis treatment used to obtain BC nanocrystals before doping with a low concentration of up to 12% in mass of magnetite. With these methods, the authors sought to align the spatial orientations of magnetic BC nanocrystals, leaving the material translucent and ideal for photonic applications.

### 3.1. Magnetic Particles Added to BC

There are several options for dopants with magnetic properties. Iron oxides and derivatives such as magnetite, maghemite, hematite and ferrites can be found in nature as ores and can also be synthesized in the laboratory [83,84]. Table 3 presents some studies that produced magnetic cellulose biomembranes, listing the respective magnetic dopants and applications.

The choice of dopant varies according to the application and objectives of each study. However, most researchers perform doping with iron oxides, especially magnetite due to its low toxicity, high magnetization, low synthesis cost, crystallinity and nanometric size [67,86,87,90,91].

Iron oxides constitute a group of compounds produced from the oxidation of this metal. The oxidation state of iron defines each of these compounds, which have different properties and are divided into 16 types [99]. Magnetite and maghemite are widely used to produce magnetic BC, as shown in Table 3. There are also reports of the use of ferrites, a type of material derived from iron oxides with the addition of metals [46,97].

Magnetite (Fe_3_O_4_) and maghemite (y-Fe_2_O_3_) are biocompatible ferromagnetic oxides which have a spinel crystalline structure and can be obtained through physical, chemical, and biological routes. The coercivity and saturation magnetization of these minerals are generally high but vary depending on the size of the crystals. One of the challenges in the synthesis of these materials is the ease of oxidation, as the particles can be oxidized with atmospheric air. For instance, one of these oxidative processes is the formation of maghemite during the synthesis of magnetite due to the oxidation of the latter during the process. It is therefore common to obtain both types of particles in certain experiments [84,100].

Another interesting feature is that when these particles are in specific nanometric sizes, forming a magnetic monodomain, they change their magnetic property to superparamagnetic, which is a characteristic that makes the material magnetize only in the presence of an external magnetic field [101].

Magnetic ferrites are derived from iron oxides associated with ions of other metals. The production of this type of material takes place at high temperatures. The most common metals are barium, cobalt, manganese, nickel, and strontium [83,102]. Ferrites can be classified according to their magnetic coercivity, i.e., ease of magnetization and demagnetization, as soft (those with greater ease of magnetization due to an external magnetic field and demagnetization in its absence) or hard (those that do not lose their magnetization and are permanent magnets) [103]. Examples of soft ferrites are manganese and nickel, whereas strontium, cobalt and barium are hard ferrites [103,104,105].

In general, particles of maghemite, magnetite and ferrites used as magnetic dopants for BC have a size on the nanometric order to ensure a better interaction with the nanometric fibers of the biopolymer. Thus, a biotechnological and magnetic BC material is produced. There are also several ways of inserting these dopants into BC fibers, such as methods that carry out the synthesis of dopants in situ and ex situ, which will be discussed in the next section.

### 3.2. Forms of BC Magnetic Doping

Table 3 demonstrates the recurrence of methods used among the cited works. The method most used is in situ co-precipitation, followed by the incorporation of previously made particles, thermal decomposition and in situ electrolysis.

With the in situ co-precipitation process, precursor compounds are adsorbed by the BC and other agents are then added, thus transforming it into a small bioreactor: a medium where the synthesis of the dopant takes place. At the end of the synthesis, the magnetic particles precipitate inside the BC nanofibers [19].

Chanthiwong et al. [82] used co-precipitation to produce magnetic nanoparticles within BC fibres. The authors left the membrane immersed in an aqueous solution containing Fe^2+^ and Fe^3+^ ions. The membrane absorbed the ions and was then immersed in an oxidizing solution, converting the metallic ions into Fe_3_O_4_ and Fe_2_O_3_ (magnetite and maghemite, respectively). Another interesting point was presented in the work by Vitta et al. [98], who used BC in its coconut gel cube form, which is a sweet commonly consumed in Asia made from the fermentation of biocellulose with coconut water, demonstrating a little more of the versatility of this material. Other works describe addition via in situ co-precipitation [16,67,85,90,91,93,95,97].

Zeng et al. [96] and Mira-Cuenca et al. [92] performed the synthesis of magnetic particles in situ using a microwave device to perform a synthesis of thermal decomposition within the membranes. Zhou et al. [88] synthesized magnetite via electrolysis within BC membranes.

Thus, several methods are available for incorporating magnetic particles into a BC matrix, which can guide other researchers who intend to obtain this type of material with different techniques and compounds capable of adapting to different contexts and goals.

Another common form of incorporation is the addition of ready-made magnetic compounds synthesized ex situ, which is described in several works [29,81,86,87,94]. For instance, Sriplai et al. [87] incorporated magnetite in BC in a very simple way. The authors immersed BC films in aqueous solutions with different proportions of a commercial ferrofluid and maintained the BC submerged in the solutions for 1 h under agitation at 80 °C to facilitate the dispersion of the magnetic particles over the membrane. At the end of the process, the authors obtained uniform magnetic films. While co-precipitation methods depend not only on the absorption of particles and reagents in the BC, but also on the synthesis of the magnetic dopant, this type of method generally depends on the absorption capacity, since the particles have previously been made.

As shown in Table 3, most researchers opted for the synthesis of co-precipitation of magnetic particles within the structure of the biomembrane, making it a bioreactor. According to Chanthiwong et al. [82], this method became popular due to its simplicity, the good distribution of particles in the BC and the possibility of adjusting the reagents. However, not all studies use this method. Salidkul et al. [81], for example, performed ex situ doping with barium ferrite. According to the authors, there is no way to carry out the safe in situ precipitation in BC, as the synthesis of these nanoparticles occurs at very high temperatures (>800 °C), which would degrade the BC membranes. There is also a discussion of the control of the amount, distribution, size, and shape of the dopant particles added to the BC in in situ and ex situ precipitation methods. Some authors claim that classic in situ co-precipitation does not enable good control over these variables, whereas others suggest adaptations and adjustments of the reaction conditions to obtain greater control over the particles [90,91,98]. Therefore, the choice of doping method depends on factors such as the nature of the dopant, degree of complexity, availability of materials and the desired application of the biomaterial.

### 3.3. Applications

One of the main advantages of magnetic cellulose (BC and VC) production is the diversity of cellulosic fibres and dopant particles that can be added [106,107]. This wide variety of options enables the production of different biotechnological materials, which can assume different shapes and applications. There are several suggestions and applications for magnetic materials derived from BC biofilms, as presented in Table 3. Most applications are in the field of electronics and materials used in medical devices, due both to the characteristics of the magnetic material incorporated and the intrinsic properties of BC [21].

There are reports of applications of magnetic BC in several fields, such as in the manufacture of enzyme and protein immobilization systems, application in the food industry, as adsorbent material and for the separation of heavy metals [81,82,86,93]. The literature indicates that, in addition to a wide variety of synthesis methods, there is also a diversity of potential applications for magnetic BC biomembranes, making this a promising material for further research and investigation [5,20,108].

Interest in the medical and pharmaceutical applications of BC is generally due to its biocompatibility [109]. By conferring magnetic characteristics to the biopolymer, a biocompatible, magnetic material is produced, which can then be used in the manufacture of various systems and devices such as systems for contrast in magnetic resonance imaging exams, tissue engineering and drug delivery [33,110]. Another important point of doping with magnetic nanoparticles, especially magnetite, is the antimicrobial effect on different viruses, bacteria, and fungi [90].

Mira-Cuenca et al. [92] developed an ink with crushed BC fibers and iron oxide nanoparticles (magnetite). With this ink, the authors demonstrated that it was possible to produce different types of drawings in the form of a film. In in vivo tests involving a muscle implant, the ink was easily identified through magnetic resonance imaging. Thus, the material performed very well as a transverse relaxation contrast agent, enabling the monitoring of surgical implants containing this dye in a less invasive way.

Another example of a medical application is found in the work by Chaabane et al. [90]. The authors modified the structure of BC and carried out magnetite precipitation in situ, forming a magnetic BC composite. The antimicrobial properties were evaluated, and the material was also used as a chemotherapeutic agent in mice with tumours. The authors observed the effectiveness of the magnetic BC composite as an antifungal and antibacterial agent and found that the composite performed well in chemotherapy treatment in mice, preventing tumour growth.

Sensors for monitoring health conditions have been also studied. In this type of application, there is interdisciplinarity with the field of electronics, which is constantly growing and directly and indirectly impacts many other fields.

### 3.4. Electro-Electronic Applications of Magnetic BC

Electro-electronics is a constantly growing field, as other scientific fields depend on electronic devices. Such devices are also found in homes and industries, making everyone’s work and life easier. Magnetic BC also has applications in this field, since many devices have parts made of magnetic materials and there is a continuous search for their optimization. Therefore, magnetic BC has been attracting the attention of researchers [111]. Indeed, the literature reports the association between magnetic BC biofilm and electronic devices such as actuators, sensors, memory storage devices, sound amplifiers and displays [20,21,29,96].

The study by Chen et al. [16] is a good example of a practical application of magnetic BC with the development of an electromagnetic motion sensor. The sensor was composed of a flexible magnetic BC tape connected to a copper coil. The ribbon was sewn onto the sleeve of a jacket, while the bobbin was sewn to the waistband. The sensor was able to monitor the shape of the movement by detecting the oscillation frequency and speed of the arm, which was in direct contact. The volunteers in this experiment ran at different speeds. The periodicity of the arm movement generated displacement of the magnetic BC tape and an oscillation in its magnetic field, thus inducing a voltage difference between the tape and copper coil. The signals were captured in waveforms by a monitor connected to the copper coil and had different amplitudes and frequencies, corresponding to each volunteer’s running speed, showing the performance of magnetic BC as a sensor. In addition to the performance shown in the prototype, the authors also listed the flexibility and biocompatibility of the magnetic BC as advantages of the material in the application of sensors of this type [112].

Sriplai et al. [97] and Marins et al. [85] also proposed the application of magnetic BC in the production of sensors. Nakajima et al. [113] stated that magnetic characteristics and flexibility are important for the applicability of modern sensors, since flexibility gives devices freer, more adaptable forms. Sriplai et al. [19] pointed out the piezoelectric properties (ability to generate electrical voltage in response to a mechanical stimulus) in magnetic BC films, which were conferred by manganese ferrites for the development of a greater variety of sensors.

Salidkul et al. [81] recommended the application of a magnetic BC produced in their study in data storage devices due to its high magnetic coercivity. Zeng et al. [96] highlighted the use of the magnetic membrane in electrical circuits and in the assembly of sound amplifiers, since magnetic BC showed good flexibility and magnetization in this study. The authors also relate this last possibility of application to a work carried out by Galland et al. [114], who produced a prototype with a magnetic VC membrane, which dispensed with the presence of a magnet inside. Another work in which the authors built an amplifier with a material similar to magnetic BC, supporting the application proposed by Wu et al. [115], was that of Tarrés et al. [116], who performed treatment on a VC extract, making its structure nanometric, which was then magnetized with magnetite.

With the great diversity of possible direct applications of magnetic BC according to its characteristics, performance in prototypes and associated with prototypes made with similar materials, magnetic BC has considerable biotechnological potential for the construction of materials and electronic devices, and should receive more attention from researchers regarding practical applications and advances in making more prototypes.

## 4. Conclusions and Future Perspectives

Biotechnological materials involving the use of BC have been widely investigated in the literature. Magnetic BC combines the unique characteristics of the biopolymer such as biocompatibility, biodegradability and renewable production, with magnetic characteristics derived from added particles such as magnetite and ferrites. These novel materials have considerable versatility in terms of production and application, with reports of applications in the fields of medicine, environmental remediation and the immobilization of compounds. There is also a growing demand for new materials in the field of electronics, as many of those currently used come from production chains that generate polluting waste. Therefore, a growing number of studies have investigated the application of magnetic BC in this field. Several studies have shown magnetic BC to be a promising material for the construction of electronic devices, such as sensors, actuators, data storage devices and sound amplifiers.

With all these unique characteristics and scientific findings of potential electro-electronic applications, magnetic BC is a biomaterial for increasingly innovative applications, enabling the production of new devices and solutions as well as reducing the use of polluting materials commonly found in electronic devices.

## Figures and Tables

**Table 1:** Comparison of properties of bacterial cellulose (BC) and vegetable cellulose (VC).

Properties	Bacterial Cellulose	Vegetable Cellulose	Reference
Purity (%)	>99	<80	[9,24]
Crystallinity degree (%)	60–90	40–78	
Fibre diameter size scale	Nanometric	Micrometric	[25,26]
Young’s modulus (GPa)	15–30	1.5–203	[6,27]
Magnetic saturation (emu/g)	0	0	[28,29]
Coercivity (kOe)	0	0	

**Table 2:** Some dopants and additives used to impart different characteristics to BC.

Dopant	Added Properties	References
Silver nanoparticles	Antimicrobial	[50,51,52,53,54]
Polypyrrole	Electrical conductivity	[13,55,56,57,58]
Polyaniline	Electrical conductivity	[59,60,61]
Graphene/Graphene oxide	Mechanical Strength and electrical conductivity	[62,63,64]

**Table 3:** Magnetic materials added to BC and respective applications.

Magnetic Dopants	Forms of BC Magnetic Doping	Saturation Magnetization of Magnetic BC (emu/g) *	Coercive Field of Magnetic BC (Oe) *	Applications	References
Magnetite	In situ co-precipitation	60.0	15	Application in nonlinear optics, clinical applications such as contrast, agents for magnetic resonance, hyperthermia and cell separation and sensors.	[85]
	Incorporation of previously made particles	41.0	27	Absorption of heavy metals	[86]
	Incorporation of previously made particles	53.6	**	Actuators, sensors, flexible data storage	[87]
	In situ electrolysis	4.2–21.2	**	Electronic and magnetic devices, enzymatic assays, drug delivery systems	[88]
	In situ co-precipitation	***	***	Tissue reconstruction	[89]
	In situ co-precipitation	23.63	0.042	Drug delivery	[90]
	In situ co-precipitation	5.14–11.56	**	Electronic devices	[67]
	In situ co-precipitation	40.57	**	Electronic devices	[91]
	Thermal decomposition	***	***	Magnetic resonance device	[92]
	In situ co-precipitation	34.07	**	Device for enzyme immobilization	[93]
	Incorporation of previously made particles	***	***	Drug delivery	[94]
	Incorporation of previously made particles	0.14	**	Optical materials	[29]
Cobalt ferrite	In situ co-precipitation	3.769–5.026	5000	Electric actuators	[95]
				Sensors	[16]
Maghemite	Thermal decomposition	60	**	Sound amplifier devices	[96]
Barium ferrite	Incorporation of previously made particles	24.1–49.5	5.31	Data storage devices, electromagnetic adsorbers	[81]
Magnetite and maghemite	In situ co-precipitation	55.0–61.0	**	Sensors, actuators, and metal adsorbents	[82]
Manganese ferrite	In situ co-precipitation	***	***	Sensors	[97]
Nickel nanoparticles	In situ co-precipitation	2.8	28	Magnetic ink and magnetic scaffolds for tissue engineering	[98]

* Measurements at room temperature. ** Values near to zero. *** The authors did not measure the values in such referenced articles.

## Data Availability

Not applicable.

## References

[1] Zhu M., Zhang J., Xu W., Xiong R., Huang C. (2023). Cellulose-based fibrous materials for self-powered wearable pressure sensor: A mini review. Cellulose.

[2] Sharma A., Thakur m., Bhattacharya M., Mandal T., Saswata G. (2019). Commercial application of cellulose nano-composites—A review. Biotechnol. Rep..

[3] Betlej I., Zakaria S., Krajewski K.J., BoruszewskIi P. (2021). Bacterial cellulose–properties and its potential application. Sains Malays.

[4] Romero-Montero A., Valencia-Bermúdez J.L., Rosas-Meléndez S.A., Núñez-Tapia I., Piña-Barba M.C., Leyva-Gómez G., Del Prado-Audelo M.L. (2023). Biopolymeric Fibrous Aerogels: The Sustainable Alternative for Water Remediation. Polymers.

[5] Campano C., Balea A., Blanco A., Negro C. (2016). Enhancement of the fermentation process and properties of bacterial cellulose: A review. Cellulose.

[6] Amorim J.D.P., Souza K.C., Duarte C.R., Duarte I.S., Ribeiro F.A.S., Silva G.S., Farias P.M.A., Sting A., Costa A.F.S., Vinhas G.M. (2020). Plant and bacterial nanocellulose: Production, properties and applications in medicine, food, cosmetics, electronics and engineering. A review. Environ. Chem. Lett..

[7] Costa A.F.S., Almeida F.C.G., Vinhas G.M., Sarubbo L.A. (2017). Production of Bacterial Cellulose by Gluconacetobacter Hansenii Using Corn Steep Liquor as Nutrient Sources. Front. Microbiol..

[8] Costa A.F.S., Silva C.J.G., Meira H.M., Amorim J.D.P., Silva I., Gomes E.S., Sarubbo L.A. (2020). Production of Paper Using Bacterial Cellulose and Residue From The Sugar and Alcohol Industry. Chem. Eng. Trans..

[9] Silva C.J.G., Medeiros A.D.M., Amorim J.D.P., Nascimento H.A., Converti A., Costa A.F.S., Sarubbo L.A. (2021). Bacterial cellulose biotextiles for the future of sustainable fashion: A review. Environ. Chem. Lett..

[10] Nascimento H.A., Amorim J.D.P., Filho L.E.P.T.M., Costa A.F.S., Sarubbo L.A., Napoleão D.C., Vinhas G.M. (2022). Production of Bacterial Cellulose with Antioxidant Additive from Grape Residue with Promising Cosmetic Applications. Polym. Eng. Sci..

[11] Albuquerque R.M.P., Meir H.M., Silva I.D.L., Silva C.J.G., Almeida F.C.G., Amorim J.D.P., Vinhas G.M., Costa A.F.S., Sarubbo L.A. (2021). Production of A Bacterial Cellulose/Poly (3-Hydroxybutyrate) Blend Activated with Clove Essential Oil For Food Packaging. Polym. Polym. Compos..

[12] Amorim J.D.P., Nascimento H.A., Silva C.J.G., Medeiros A.D.M., Silva I.D.L., Costa A.F.S., Vinhas G.M., Sarubbo L.A. (2021). Obtainment Of Bacterial Cellulose With Added Propolis Extract For Cosmetic Applications. Polym. Eng. Sci..

[13] Huo Y., Guo D., Yang J., Chang Y., Wang B., Mu C., Xiang J., Nie A., Zhai K., Xue T. (2022). Multifunctional Bacterial Cellulose Nanofibers/Polypyrrole (Ppy) Composite Films for Joule Heating and Electromagnetic Interference Shielding. ACS Appl. Electron. Mater..

[14] Moon R.J., Martini A., Nairn J., Simonsen J., Youngblood J. (2011). Cellulose nanomaterials review: Structure, properties and nanocomposites. Chem. Soc. Rev..

[15] Urbina L., Corcuera M.A., Gabilondo N., Eceiza A., Retegi A. (2021). A review of bacterial cellulose: Sustainable production from agricultural waste and applications in various fields. Cellulose.

[16] Chen K., Li Y., Du Z., Hu S., Huang J., Shi Z., Su B., Yang G. (2022). Cofe_2_O_4_ Embedded Bacterial Cellulose for Flexible, Biodegradable, and Self-Powered Electromagnetic Sensor. Nano Energy.

[17] Safarik I., Safarikova M. (2009). Magnetic nano-and microparticles in biotechnology. Chem. Zvesti.

[18] Merazzo K.J., Lima A.C., Rincón-Iglesias M., Fernandes L.C., Pereira N., Lanceros-Mendez S., Martins P. (2021). Magnetic materials: A journey from finding north to an exciting printed future. Mater. Horiz..

[19] Sriplai N., Pinitsoontorn S. (2020). Bacterial cellulose-based magnetic nanocomposites: A review. Carbohydr. Polym..

[20] Kumar A., Sood A., Han S.S. (2022). Potential of magnetic nano cellulose in biomedical applications: Recent Advances. Biomater. Polym. Horiz..

[21] Jin K., Jin C., Wu Y. (2022). Synthetic Biology-Powered Microbial Co-Culture Strategy and Application of Bacterial Cellulose-Based Composite Materials. Carbohydr. Polym..

[22] Singhania R.R., Patel A.K., Tsai M.L., Chen C.W., Dong C.D. (2021). Genetic modification for enhancing bacterial cellulose production and its applications. Bioengineered.

[23] Yu C., Sinclair R. (2014). Natural textile fibres: Vegetable fibres. Textiles and Fashion.

[24] Wang J., Tavakoli J., Tang Y. (2019). Bacterial cellulose production, properties and applications with different culture methods–A review. Carbohydr. Polym..

[25] Medeiros A.D.L.M., Silva C.J.G., Amorim J.D.P., Durval I.J.B., Costa A.F.S., Sarubbo L.A. (2022). Oily Wastewater Treatment: Methods, Challenges, and Trends. Processes.

[26] Wasin M., Shi F., Liu J., Khan M.R., Farooq A., Sanbha N., Alfred M., Xin L., Yajun C., Zhao X. (2021). Extraction of cellulose to progress in cellulosic nanocomposites for their potential applications in supercapacitors and energy storage devices. J. Mater. Sci..

[27] Manimaran P., Pillai G.P., Vignesh V., Prithiviraj M. (2020). Characterization of natural cellulosic fibers from Nendran Banana Peduncle plants. Int. J. Biol. Macromol..

[28] Sriplai N., Mongkolthanaruk W., Eichhorn S.J., Pinitsoontorn S. (2020). Magnetic bacterial cellulose and carbon nanofiber aerogel by simple immersion and pyrolysis. J. Mater. Sci..

[29] Zhang X., Kang S., Adstedt K., Kim M., Xiong R., Yu J., Chen X., Zhao X., Ye C., Tsukruk V.V. (2022). Uniformly Aligned Flexible Magnetic Films from Bacterial Nanocelluloses for Fast Actuating Optical Materials. Nat. Commun..

[30] Torres F.G., Commeaux S., Troncoso O.P. (2012). Biocompatibility of bacterial cellulose-based biomaterials. J. Funct. Biomater..

[31] Portela R., Leal C.R., Pedro L. (2019). Minireview Bacterial cellulose: A versatile biopolymer for wound dressing applications. Microb. Biotechnol..

[32] Liu W., Du H., Zhang M., Liu K., Liu H., Xie H., Zhang X., Si C. (2020). Bacterial cellulose-based composite scaffolds for biomedical applications: A review. ACS Sustain. Chem. Eng..

[33] Eslahi N., Mahmoodi A., Mahmoudi N., Zandi N., Simchi A. (2019). Processing and properties of nanofibrous bacterial cellulose-containing polymer composites: A review of recent advances for biomedical applications. Polym. Rev. (Phila Pa).

[34] Esa F., Tasirin S.M., Rahman N.A. (2014). Overview of Bacterial Cellulose Production and Application. Ital. Oral Surg..

[35] Watanabe K., Tabuchi M., Morinaga Y., Yoshinaga F. (1998). Structural features and properties of bacterial cellulose produced in agitated culture. Cellulose.

[36] Sperotto G., Stasiak L.G., Godoi J.P.M.G., Gabiatti N.C., Souza S.S. (2021). A review of culture media for bacterial cellulose production: Complex, chemically defined and minimal media modulations. Cellulose.

[37] Raut M.P., Asare E., Syed Mohamed S.M.D., Amadi E.N., Roy I. (2023). Bacterial Cellulose-Based Blends and Composites: Versatile Biomaterials for Tissue Engineering Applications. Int. J. Mol. Sci..

[38] Hestrin S., Schramm M. (1954). Synthesis of cellulose by *Acetobacterxylinum*: II. Preparation of freeze—Dried cells capable of polymerized glucose to cellulose. Biochem. J..

[39] Kurosumi A., Sasaki C., Yamashita Y., Nakamura Y. (2019). Utilization of various fruit juices as carbon source for production of bacterial cellulose by *Acetobacter xylinum* NBRC 13693. Carbohydr. Polym..

[40] Duarte E.B., Andrade F.K., Lima H.L.S., Nascimento E.S., Carneiro M.J.M., Borges M.F., Luz E.P.C.G., Chagas B.S., Rosa M.F. (2019). Documentos186—Celulose Bacteriana Propriedades, Meios Fermentativos E Aplicações.

[41] Ul-Islam M., Ullah M.W., Khan S., Park J.K. (2017). Strategies for cost-effective and enhanced production of bacterial cellulose. Int. J. Biol. Macromol..

[42] Ul-Islam M., Ullah M.W., Khan S., Park J.K. (2020). Production of bacterial cellulose from alternative cheap and waste resources: A step for cost reduction with positive environmental aspects. Korean J. Chem. Eng..

[43] Hussain Z., Sajjad W., Khan T., Wahid F. (2019). Production of bacterial cellulose from industrial wastes: A review. Cellulose.

[44] Hu W., Chen S., Yang J., Li Z., Wang H. (2014). Functionalized bacterial cellulose derivatives and nanocomposites. Carbohydr. Polym..

[45] Zhong C. (2020). Industrial-scale production and applications of bacterial cellulose. Front. Bioeng. Biotechnol..

[46] Torres F.G., Arroyo J.J., Troncoso O.P. (2019). Bacterial cellulose nanocomposites: An all-nano type of material. Mater. Sci. Eng. C.

[47] Nascimento H.A., Amorim J.D.P., Silva C.J.G., Medeiros A.D.L.M., Costa A.F.S., Napoleão D.C., Vinhas G.M., Sarubbo L.A. (2022). Influence of gamma irradiation on the properties of bacterial cellulose produced with concord grape and red cabbage extracts. Curr. Res. Biotechnol..

[48] Amarasekara A.S., Wang D., Grady T.L. (2020). A comparison of kombucha SCOBY bacterial cellulose purification methods. Appl. Sci..

[49] Cazón P., Vázquez M. (2021). Improving bacterial cellulose films by ex-situ and *in-situ* modifications: A review. Food Hydrocoll..

[50] Barud H.S., Regiani T., Marques R.F.C., Lustri W.R., Messaddeq Y., Ribeiro S.J.L. (2011). Antimicrobial Bacterial Cellulose-Silver Nanoparticles Composite Membranes. J. Nanomater..

[51] Zhao H., Zhang L., Zheng S., Chai S., Wei J., Zhong L., He Y., Xue J. (2022). Bacteriostatic Activity and Cytotoxicity of Bacterial Cellulose-Chitosan Film Loaded with In-Situ Synthesized Silver Nanoparticles. Carbohydr. Polym..

[52] Audtarat S., Hongsachart P., Dasri T., Chio-Srichan S., Soontaranon S., Wongsinlatam W., Sompech S. (2022). Green synthesis of silver nanoparticles loaded into bacterial cellulose for antimicrobial application. Nanocomposites.

[53] Anwar Y., Ul-Islam M., Mohammed Ali H.S.H., Ullah I., Khalil A., Kamal T. (2022). Silver impregnated bacterial cellulose-chitosan composite hydrogels for antibacterial and catalytic application. J. Mater. Res. Technol..

[54] Rakhimova B., Kudaibergenov K., Sassykova L., Spanova G., Aknazarov S., Tulepov M. (2022). Preparation of Composites of Antibacterial Materials Based on Bacterial Cellulose and Silver Nanoparticles for Wound Healing. Int. J. Nanosci. Nanotechnol..

[55] Müller D., Rambo C.R., Recouvreu D.O.S., Porto L.M., Barra G.M.O. (2011). Chemical in Situ Polymerization of Polypyrrole on Bacterial Cellulose Nanofibers. Synth. Met..

[56] Li S., Huang D., Yang J., Zhang B., Zhang X., Yang G., Wang M., Shen Y. (2014). Freestanding Bacterial Cellulose–Polypyrrole Nanofibres Paper Electrodes for Advanced Energy Storage Devices. Nano Energy.

[57] Krathumkhet N., Imae T., Paradee N. (2021). Electrically controlled transdermal ibuprofen delivery consisting of pectin-bacterial cellulose/polypyrrole hydrogel composites. Cellulose.

[58] Fraser S.A., Van Zyl W.E. (2022). In situ polymerization and electrical conductivity of polypyrrole/cellulose nanocomposites using Schweizer’s reagent. RSC Adv..

[59] Lee B.H., Kim H.J., Yang H.S. (2012). Polymerization of Aniline on Bacterial Cellulose and Characterization Of Bacterial Cellulose/Polyaniline Nanocomposite Films. Curr. Appl. Phys..

[60] Rana A.K., Scarpa F., Thakur V.K. (2022). Cellulose/polyaniline hybrid nanocomposites: Design, fabrication, and emerging multidimensional applications. Ind. Crops Prod..

[61] Hosseini H., Mousavi S.M., Wurm F.R., Goodarzi V. (2021). Display of hidden properties of flexible aerogel based on bacterial cellulose/polyaniline nanocomposites with helping of multiscale modeling. Eur. Plym. J..

[62] Guan F., Chen S., Sheng N., Chen Y., Yao J., Pei Q., Wang H. (2019). Mechanically Robust Reduced Graphene Oxide/Bacterial Cellulose Film Obtained Via Biosynthesis for Flexible Supercapacitor. Chem. Eng. J..

[63] Gabryś T., Fryczkowska B., Fabia J., Biniaś D. (2022). Preparation of an Active Dressing by In Situ Biosynthesis of a Bacterial Cellulose–Graphene Oxide Composite. Polymers.

[64] Zhang L., Yu Y., Zheng S., Zhong L., Xue J. (2021). Preparation and properties of conductive bacterial cellulose-based graphene oxide-silver nanoparticles antibacterial dressing. Carbohydr. Polym..

[65] Fernandes I.A.A., Pedro A.C., Ribeiro V.R., Bortolini D.G., Ozaki M.S.C., Maciel G.M., Haminiuk C.W.I. (2020). Bacterial cellulose: From production optimization to new applications. Int. J. Biol. Macromol..

[66] Gregory D.A., Tripathi L., Fricker A.T.R., Asare E., Orlando I., Raghavendran V., Roy I. (2021). Bacterial cellulose: A smart biomaterial with diverse applications. Mater. Sci. Eng. R Rep..

[67] Usawattanakul N., Torgbo S., Sukyai P., Khantayanuwong S., Puangsin B., Srichola P. (2021). Development of nanocomposite film comprising of Polyvinyl Alcohol (PVA) incorporated with bacterial cellulose nanocrystals and magnetite nanoparticles. Polymers.

[68] Shu Y., Bai Q., Fu G., Xiong Q., Li C., Ding H., Uyama H. (2020). Hierarchical Porous Carbons from Polysaccharides Carboxymethyl Cellulose, Bacterial Cellulose, and Citric Acid for Supercapacitor. Carbohydr. Polym..

[69] Bai Q., Shen Y., Asoh T.A., Li C., Dan Y., Uyama H. (2020). Controlled preparation of interconnected 3D hierarchical porous carbons from bacterial cellulose-based composite monoliths for supercapacitors. Nanoscale..

[70] Wang Z., Wang K., Yao X., Jiang J., Wang M., Yuan S. (2023). Ultrasound-assisted preparation of Fe(OH)_3_@bacterial cellulose aerogel for efficient removal of organic contamination in water. Appl. Surf. Sci..

[71] Choi S.M., Shin E.J. (2020). The Nanofication and Functionalization of Bacterial Cellulose and Its Applications. Nanomaterials.

[72] Safae M., Taran M. (2021). Preparation of Bacterial Cellulose Fungicide Nanocomposite Incorporated with MgO Nanoparticles. J. Polym. Environ..

[73] Mirtalebi S.S., Almasi H., Khaledabad M.A. (2019). Physical, morphological, antimicrobial and release properties of novel MgO-bacterial cellulose nanohybrids prepared by *in-situ* and *ex-situ* methods. Int. J. Biol. Macromol..

[74] Coey J.M. (2010). Magnetism and Magnetic Materials.

[75] Shackelford J.F. (2008). Introdução à Ciencia Dos Materiais.

[76] Spaldin N.A. (2012). Magnetic Materials: Fundamentals and Applications.

[77] Callister W.D., Rethwisch D.G. (2016). Ciência E Engenharia De Materiais: Uma Introdução.

[78] Sheng P., Wang B., Li R. (2018). Flexible Magnetic Thin Films and Devices. J. Semicond..

[79] Fatima H., Charinpanitkul T., Kim K.S. (2021). Fundamentals to Apply Magnetic Nanoparticles for Hyperthermia Therapy. Nanomaterials.

[80] Kianfar E. (2021). Magnetic Nanoparticles in Targeted Drug Delivery: A Review. J. Supercond. Nov. Magn..

[81] Salidkul N., Mongkolthanaruk W., Faungnawakij K., Pinitsoontorn S. (2021). Hard magnetic membrane based on bacterial cellulose–barium ferrite nanocomposites. Carbohydr. Polym..

[82] Chanthiwong M., Mongkolthanaruk W., Eichhorn S.J., Pinitsoontorn S. (2020). Controlling the processing of co-precipitated magnetic bacterial cellulose/iron oxide nanocomposites. Mater. Des..

[83] Houbi A., Aldashevich Z.A., Atassi Y., Bagasharova Telmanovna Z., Saule M., Kubanych K. (2021). Microwave Absorbing Properties of Ferrites and Their Composites: A Review. J. Magn. Magn. Mater..

[84] Niculescu A.G., Chircov C., Grumezescu A.M.I. (2021). Magnetite Nanoparticles: Synthesis Methods–A Comparative Review. Methods.

[85] Marins J.A., Soares B.G., Barud H.S., Ribeiro S.J. (2013). L Flexible magnetic membranes based on bacterial cellulose and its evaluation as electromagnetic interference shielding material. Materi. Sci. Eng C.

[86] Zhu H., Jia S., Wan T., Jia Y., Yang H., Li J., Yan L., Zhong C. (2011). Biosynthesis of spherical Fe_3_O_4_/bacterial cellulose nanocomposites as adsorbents for heavy metal ions. Carbohydr. Polym..

[87] Sriplai N., Mongkolthanaruk W., Eichhorn S.J., Pinitsoontorn S. (2018). Magnetically responsive and flexible bacterial cellulose membranes. Carbohydr. Polym..

[88] Zhou J., Li R., Liu S., Li Q., Zhang L., Zhang L., Guan J. (2009). Structure and magnetic properties of regenerated cellulose/Fe_3_O_4_ nanocomposite films. J. Appl. Polym. Sci..

[89] Pavón J.J., Allain J.P., Verma D., Echeverry-Rendón M., Cooper C.L., Reece L.M., Shetty A.R., Tomar V. (2019). In situ Study Unravels Bio-Nanomechanical Behavior in a Magnetic Bacterial Nano-cellulose (MBNC) Hydrogel for Neuro-Endovascular Reconstruction. Macromol. Biosci..

[90] Chaabane L., Chahdoura H., Mehdaoui R., Snoussi M., Beyou E., Lahcini M., Baouab M.H.V. (2020). Functionalization of developed bacterial cellulose with magnetite nanoparticles for nanobiotechnology and nanomedicine applications. Carbohydr. Polym..

[91] Yingkamhaeng N., Intapan I., Sukyai P. (2018). Fabricação e caracterização de nanocelulose bacteriana superparamagnética funcionalizada usando síntese in situ assistida por ultra-som. Fibers Polym..

[92] Mira-Cuenca C., Meslier T., Roig-Sanchez S., Laromaine A., Roig A. (2021). Patterning Bacterial Cellulose Films with Iron Oxide Nanoparticles and Magnetic Resonance Imaging Monitoring. ACS Appl. Polym. Mater..

[93] Qiao W., Zhang Z., Qian Y., Xu L., Guo H. (2022). Bacterial laccase immobilized on a magnetic dialdehyde cellulose without cross-linking agents for decolorization. Colloids Surf. A Physicochem. Eng. Asp..

[94] da Rosa Salles T., da Silva Bruckamann F., Viana A.R., Krause L.M.F., Mortari S.R., Rhoden C.R.B. (2022). Magnetic nanocrystalline cellulose: Azithromycin adsorption and in vitro biological activity against melanoma cells. J. Polym. Environ..

[95] Olsson R.T., Azizi M.A.S., Salazar-Alvarez G., Belova L., Ström V., Berglund L.A., Ikkala O., Nogués J., Gedde U.W. (2010). Making flexible magnetic aerogels and stiff magnetic nanopaper using cellulose nanofibrils as templates. Nat. Nanotechnol..

[96] Zeng M., Laromaine A., Feng W., Levkin P.A., Roig A. (2014). Origami magnetic cellulose: Controlled magnetic fraction and patterning of flexible bacterial cellulose. J. Mater. Chem. C.

[97] Sriplai N., Mangayil R., Pammo A., Santala V., Tuukkanen S., Pinitsoontorn S. (2020). Enhancing piezoelectric properties of bacterial cellulose films by incorporation of MnFe_2_O_4_ nanoparticles. Carbohydr. Polym..

[98] Vitta S., Drillon M., Derory A. (2010). Magnetically responsive bacterial cellulose: Synthesis and magnetic studies. J. Appl. Phys..

[99] Silva M.F., Pineda E.A.G., Bergamasco R. (2015). Aplicação de óxidos de ferro nanoestruturados como adsorventes e fotocatalisadores na remoção de poluentes de águas residuais. Quím. Nova.

[100] Shokrollahi H. (2017). A Review of The Magnetic Properties, Synthesis Methods and Applications of Maghemite. J. Magn. Magn. Mater..

[101] Vaewbundit S., Siriphannon P. (2022). Soft solution growth of magnetite-maghemite nanocrystals in crosslinked chitosan templates and their superparamagnetic properties. Nanocomposites.

[102] Fontanive V.C.P., Khalil N.M., Cotica L.F., Mainardes R.M. (2014). Aspectos Físicos e Biológicos de Nanopartículas de Ferritas Magnéticas. Rev. Ciênc. Farm. Básica E Apl..

[103] Sagayaraj R., Aravazhi S., Chandrasekaran G. (2021). Review on Structural and Magnetic Properties of (Co–Zn) Ferrite Nanoparticles. Int. Nano Lett..

[104] Amiri M., Salavati-Niasari M., Akbari A. (2019). Magnetic Nanocarriers: Evolution of Spinel Ferrites for Medical Applications. Adv. Colloid Interface Sci..

[105] Narang S.B., Pubby K. (2021). Nickel Spinel Ferrites: A Review. J. Magn. Magn. Mater..

[106] Akhlaghi N., Najafpour-Darzi G. (2021). Manganese Ferrite (Mnfe_2_O_4_) Nanoparticles: From Synthesis to Application-A Review. J. Ind. Eng. Chem..

[107] Nypelö T. (2022). Magnetic Cellulose: Does Extending Cellulose Versatility with Magnetic Functionality Facilitate Its Use In Devices?. J. Mater. Chem. C.

[108] Andriani D., Apriyana A.Y., Karina M. (2020). The optimization of bacterial cellulose production and its applications: A review. Cellulose.

[109] Moscovici M. (2015). Present and Future Medical Applications of Microbial Exopolysaccharides. Front Microbiol..

[110] Aditya T., Allain J.P., Jaramillo C., Restrepo A.M. (2022). Surface modification of bacterial cellulose for biomedical applications. Int. J. Mol. Sci..

[111] Guan F., Guo C.F. (2021). Flexible, high-strength, and porous nano-nano composites based on bacterial cellulose for wearable electronics: A review. Soft Sci..

[112] Choi S.M., Rao K.M., Zo S.M., Shin E.J., Han S.S. (2022). Bacterial Cellulose and Its Applications. Polymers.

[113] Nakajima T., Fujio Y., Sugahara T., Tsuchiya T. (2022). Flexible Ceramic Film Sensors for Free-Form Devices. Sensors.

[114] Galland S., Andersson R.L., Salajková M., Ström V., Olsson R.T., Berglund L.A. (2013). Cellulose Nanofibers Decorated with Magnetic Nanoparticles–Synthesis, Structure and use in Magnetized High Toughness Membranes for a Prototype Loudspeaker. J. Mater. Chem. C.

[115] Wu J., Zheng Y., Song W., Luan J., Wen X., Wu Z., Chen X., Wang Q., Guo S. (2014). In Situ Synthesis of Silver-Nanoparticles/Bacterial Cellulose Composites for Slow-Released Antimicrobial Wound Dressing. Carbohydr. Polym..

[116] Tarrés Q., Deltell A., Espinach F.X., Pèlach M.À., Delgado-Aguilar M., Mutjé P. (2017). Magnetic Bionanocomposites from Cellulose Nanofibers: Fast, Simple and Effective Production Method. Int. J. Biol. Macromol..

